# Ureteral Guidewire Looping and Entrapment above an Impacted Ureter Stone

**DOI:** 10.1155/2019/4103523

**Published:** 2019-10-17

**Authors:** Aikaterini Tsionga, Anastasios Anastasiadis, Wilbert Fana Mutomba, Dimitrios Memmos, Ioannis Vakalopoulos, Georgios Dimitriadis

**Affiliations:** 1st Department of Urology, Aristotle University of Thessaloniki, Ethnikis Aminis 41, 54635 Thessaloniki, Greece

## Abstract

We present a rare case of a hydrophilic guidewire looping and entrapment in the ureter of a patient with an impacted stone in the proximal ureter during a simple double-J stent insertion. Looping of guidewire is a rare complication in urology and only few cases have been described. In that case, release and removal of the entrapped guidewire was possible only after one step fragmentation of the stone with laser lithotripsy.

## 1. Introduction

Guidewires play a significant role in many medical specialties [1]. They were initially developed for vascular procedures [2], but are also nowadays an essential tool in endourology. Although they are used by urologists to perform various advanced procedures, such as to insert a ureteral stent, to dilate the ureter or gain percutaneous access to the kidney, they are frequently associated with potential complications. Bleeding, kinking, false passages, and perforation are some of the usual complications [3]. Looping, usually combined with knotting, is a situation more commonly described during intravascular application of guidewires [1, 4]. Nevertheless, looping above the stone and entrapment of a guidewire inside the ureter is an extremely rare complication. There is only one case in the literature, where the authors present looping of a guidewire below the stone during rigid ureteroscopy and laser lithotripsy [5].

## 2. Case Presentation

**Figure 1:**
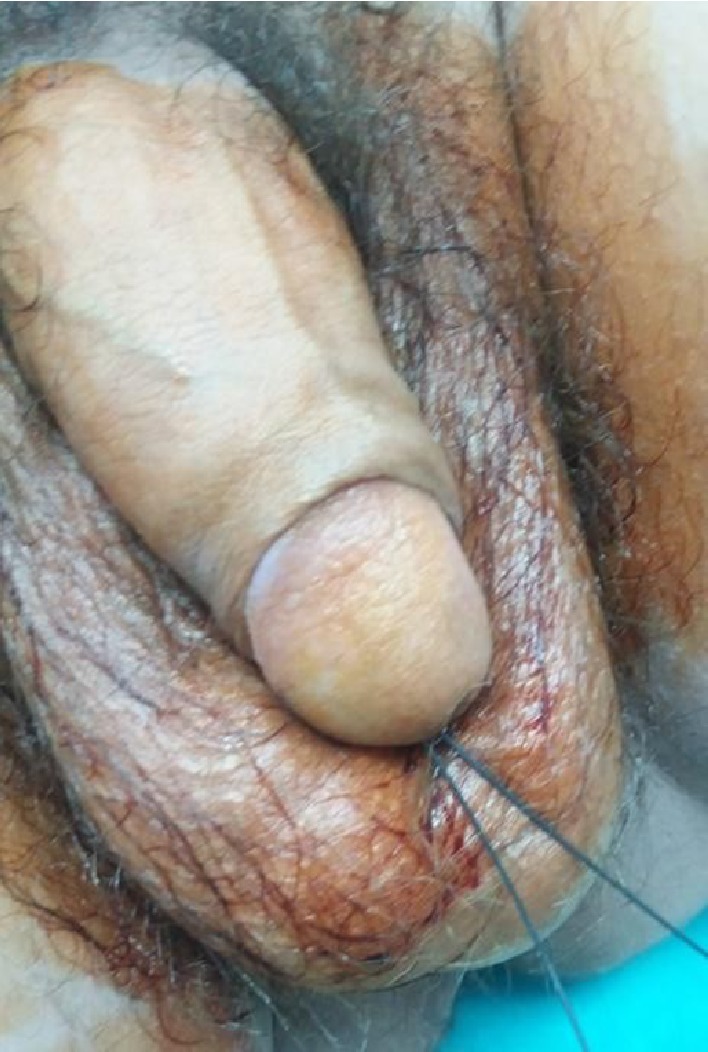
Both parts of the guidewire protruding out of the urethral meatus.

**Figure 2:**
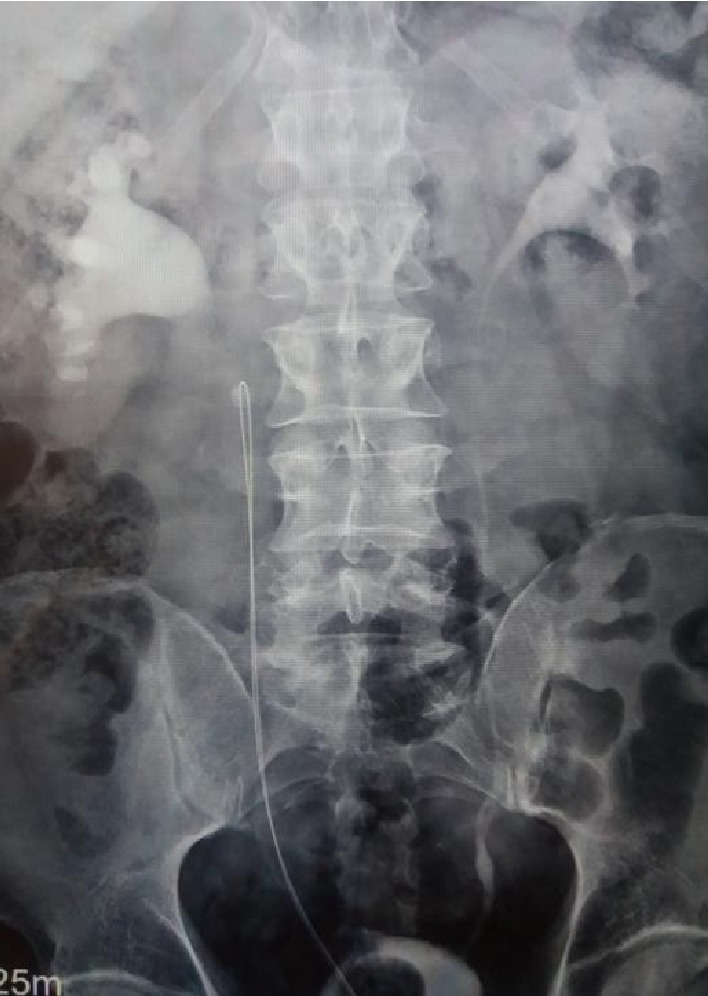
IVU reveals a right proximal ureteral obstructing stone and the guidewire looping above it.

A 57-year-old man was referred to our department by a district hospital, after a previous unsuccessful effort of a double-J ureteral stent placement without real-time fluoroscopy. The patient arrived at the emergency department with both parts of a guidewire (Terumo RadiofocusR Guidewire, 180 cm) protruding out of his urethral meatus (Figure 1). The intravenous urography (IVU), which had been performed at the district hospital after the failed stent insertion, revealed a right proximal ureteral obstructing stone with approximate maximal diameter 9 mm (9 × 4 × 6 mm) and the guidewire looping above it (Figure 2). IVU was performed instead of CT urography due to unavailability of CT urography at the referring center. The admission laboratory tests revealed no leukocytosis (WBC 10900 × 10^3^/*μ*L) and normal serum creatinine values (Cre 0.85 mg/dL). Rest of laboratory exams were normal. Also patient was in a good general clinical status.

Our first step was to pull gently one end of the guidewire under fluoroscopic guidance and local anesthesia, but our effort was unsuccessful. Next, we inserted another hybrid guidewire (Sensor™ Straight Tip, Boston Scientific), which could not bypass the stone and was looping under it. The retrograde urography showed full stop of the contrast at the level of the stone and no extravasation was revealed. Since the patient had no signs of infection, we decided to continue our handling under general anesthesia, so we could inspect the ureter with the semirigid ureteroscope (Karl Storz, 27002L, 43 cm length, 9.5 Fr). The ureteroscope was advanced easily up to the stone. The guidewire had initially perforated the mucosa of the ureter under the impacted stone, was looping intraluminally above the stone, returned submucosally and finally passed intraluminally to get out of the ureter orifice and out of the meatus. Subsequently, we performed an active stone removal via laser lithotripsy (Dornier. Medilas H Solvo, laser fiber 600 *μ*m). The entrapped guidewire was released after fragmentation of the stone and was easily removed from the ureter. Finally, a 7F-28 cm open-end double-J stent was placed over the second guidewire fluoroscopically. The patient was discharged the next day, with no complications after the ureteroscopy and the entrapped guidewire removal. The double-J was removed after two weeks under local anesthesia without any adverse events.

## 3. Discussion

As was mentioned before, guidewires are a frequently used tool that allows urologists to perform a great variety of endoscopic procedures, many times challenging and difficult ones. But, as with any tool in medicine, training is necessary to apply guidewires with safety and should be used by someone experienced [1]. There are basic principles and tips when using endourology tools, which could prevent undesirable complications.

In our case, the first attempt of double-J placement at the district hospital had been performed without fluoroscopic guidance. If the motion of the guidewire was monitored by fluoroscopy, looping direction might have been avoided. In addition, the length of the guidewire (180 cm, use in vascular surgery procedures) made it easier to form a loop inside the ureter, to come out of the ureteral orifice and out of the urethral meatus. Furthermore, if excessive force is applied when inserting the guidewire, the tip may perforate the wall and progress submucosally or advance outside the ureter. Especially, if the guidewire is forced blindly without fluoroscopy, it is difficult for the operator to realize in time the false route. Gentle maneuvers under fluoroscopy guidance and repositioning of the guidewire when operator feels resistance are important for a safe stent placement [1].

Also, when the urologist deals with impacted stones, needs to be aware that the ureteral wall at the level of the obstruction is oedematous and the guidewire may traumatize and perforate the ureteral wall even with the appliance of little force by the surgeon.

Guidewires can be found in various sizes, materials, tip design, and rigidity and each one has been developed for specific clinical use. They are used for achieving safe access to any part of the urinary system and as a guide over which stents and catheters can be passed. Guidewires with an atraumatic, flexible, and hydrophilic tip are appropriate for passing over obstructions in the urinary tract, while a wire with stiff shaft is required to place a stent over it [6]. Hybrid wires (nitilol guidewire with hydrophilic tip) seem to be the most preferable choice for safe access [7]. Different types of guidewires should be available at urology departments and the operators should be familiarized with them, as the selection of the appropriate guidewire is crucial for the success of the procedure used for.

Finally, it is very important to mention the necessity of nephrostomy tube insertion in case of urinary tract infection or elevated serum creatinine levels, before any endourological procedures. In our case, our therapeutic plan was at first to inspect the ureter with the semirigid ureteroscope. The lower part of the ureter was sufficiently large and the scope passed easily. We need to mention that we were without a normal placed guidewire, due to complete obstruction at the level of the stone. Our exit strategy, in case of unsuccessful ureterscopy, would be at the same session, the placement of a nephrostomy tube, a procedure that in our hospital is carried out by urologists. We made the same case presentation as an eposter in Berlin during 9th International Meeting “Challenges in Endourology” [8].

## 4. Conclusion

Many complications may occur during stent insertion with the aid of a guidewire. Surgeon's experience, adherence to the basic principles of endourology such as continuous imaging and appliance of little force, suspicion of impacted stone and availability of different types of guidewires are important factors to avoid various uncomfortable and difficult situations during endoscopic procedures.
